# α-Crystalline Domains and Intrinsically Disordered Regions Can Work in Parallel to Induce Accumulation of MBD6 at Chromocenters in *Arabidopsis thaliana*

**DOI:** 10.3390/epigenomes8030033

**Published:** 2024-08-28

**Authors:** Brandon A. Boone, Cristy P. Mendoza, Noah J. Behrendt, Steven E. Jacobsen

**Affiliations:** 1 Department of Molecular, Cell and Developmental Biology, University of California Los Angeles, Los Angeles, CA 90095, USA; boonebrandon25@gmail.com (B.A.B.); cristypam@gmail.com (C.P.M.); njbehrendt407@g.ucla.edu (N.J.B.); 2 Eli and Edyth Broad Center of Regenerative Medicine and Stem Cell Research, University of California Los Angeles, Los Angeles, CA 90095, USA; 3 Department of Biological Chemistry, University of California Los Angeles, Los Angeles, CA 90095, USA; 4 Howard Hughes Medical Institute (HHMI), University of California, Los Angeles, CA 90095, USA

**Keywords:** DNA methylation, protein accumulation, α-crystalline domains, intrinsically disordered regions

## Abstract

Proteins are localized and concentrated at cellular and genomic locations for specific and efficient functions. Efforts to understand protein accumulation in eukaryotic organisms have primarily focused on multivalent interactions between intrinsically disordered regions (IDRs) as mediators of protein condensation. We previously showed that α-crystalline domain (ACD) proteins 15 (ACD15) and 21 (ACD21) were required for multimerization and the accumulation of gene-silencing methyl-CpG-binding domain protein 6 (MBD6) at chromocenters in *Arabidopsis thaliana*. Here, we demonstrate that ACDs and IDRs can act as parallel mechanisms, facilitating higher-order MBD6 assemblies. Using human IDRs known to be important for protein accumulation, we replicated and enhanced the accumulation of MBD6 at chromocenters. In addition, IDRs fused to MBD6 could substitute for ACD function and partially reconstitute the MBD6 gene-silencing function. However, the accumulation of MBD6 by IDRs still required ACD15 and ACD21 for full effect. These results establish that ACD-mediated protein accumulation is a mechanism that can function similarly to and together with IDR-mediated mechanisms.

## 1. Introduction

The efficient accumulation of proteins at the appropriate density and cellular location is critical to the function of many cellular pathways. Recent discoveries of phase-separated compartments in plant and animal nuclei, formed by density-dependent protein-protein interactions, highlight the link between protein accumulation and function [1,2,3,4,5]. These supramolecular assemblies can be formed through multivalent interactions between the intrinsically disordered regions (IDRs) of proteins which are thought to function similarly to polymer-polymer interactions [5,6]. In addition, IDRs often contain small regions called short linear motifs (SLiMs) that interact with the structured domains of other proteins to facilitate protein complex formation [7,8,9,10]. 

We recently discovered a novel, chaperone-mediated, protein accumulation mechanism in the plant model organism *Arabidopsis thaliana* [11]. Methyl-CpG-binding domain protein 6 (MBD6) (as well as its functionally redundant homolog MBD5) hyper-accumulates at methylated CG (meCG) sites within pericentromeric heterochromatin, forming nuclear foci that overlap with nuclear compartments called chromocenters [11,12,13,14]. The high-level accumulation of MBD6 and its gene silencing function require its specific complex members α-crystalline domain protein 15 (ACD15) and 21 (ACD21). ACD15 and ACD21 can also phase-separate in vitro, suggesting that ACD-mediated accumulation may function similarly to IDR-driven phase-separation mechanisms [15]. 

ACD15 and ACD21 share homology with small heat-shock proteins (sHSPs) that directly regulate protein homeostasis from plants to humans. sHSPs bind hydrophobic patches of proteins and interact with J-domain proteins and HSP70 to assist in the disaggregation or refolding of proteins [16,17,18,19,20,21]. Many sHSPs are known to form dynamic oligomeric assemblies. However, unlike chaotic, IDR-driven protein assemblies, sHSPs utilize a central, structured α-crystalline domain (ACD) flanked by two small disordered regions on the N- and C-termini to form ordered oligomeric complexes [16,17,18,19,20,21]. In these sHSP oligomeric assemblies, the α-crystalline domains form dimers that interact with other dimers through the N- and C-terminal disordered regions, acting as interaction sites [16,20,21,22]. We found that ACD15 and ACD21 utilize this oligomerization capacity to facilitate the accumulation of the MBD5/6 complex at meCG sites within the pericentromeric heterochromatin-creating nuclear MBD6 foci [11]. 

The MBD5/6 complex also contains the J-domain containing protein SILENZIO (SLN), which interacts with the chaperone protein HSP70 [11,14]. Consistent with the known roles of sHSPs, ACD15 and ACD21 specifically bridge the interaction between MBD5/6 and SLN while mediating the accumulation of MBD5/6 [16]. The *silenzio*, *mbd5 mbd6*, *acd15*, and *acd21* mutants all have similar gene-silencing mutant defects, showing upregulation of the same collection of DNA-methylated genes and transposons [11]. Fluorescence Recovery After Photobleaching (FRAP) experiments also showed that all of the MBD5/6 complex members are highly mobile when forming nuclear foci at chromocenters [11]. However, the loss of SLN led to a significant decrease in MBD5/6 complex accumulation at the chromocenters and a decrease in mobility [11]. These data suggest a model in which the multimerization and high-level accumulation of MBD5/6 at meCG sites are uniquely necessary aspects of their gene-silencing functions. However, gene-silencing by MBD5/6 and the novel, ACD-mediated accumulation mechanism are still not fully understood.

Because of the similarity between IDR and ACD15/21-mediated protein oligomerization, we sought to compare these mechanisms in the context of the MBD5/6 complex. Using MBD6 chromocenter localization as a measure of protein accumulation, we created chimeric MBD6 proteins containing human or plant IDRs and showed that they could synthetically hyper-accumulate MBD6 at the chromocenters [23,24]. We then removed ACD15 and ACD21 or the MBD6 StkyC domain required for interaction with ACD15 and ACD21, leading to an inability of the MBD6-IDR chimeric proteins to hyper-accumulate at chromocenters, with only the strongest IDRs maintaining nuclear foci. However, the MBD6-IDR constructs that did maintain nuclear foci without ACD15 and ACD21 also retained some gene-silencing capacities. These results suggest that IDRs and ACD proteins act as parallel mechanisms to mediate protein accumulation and further support protein accumulation as an important aspect of the MBD5/6 complex’s gene-silencing mechanism. 

## 2. Results

### 2.1. Human IDRs Can Stimulate the Accumulation of MBD6 Together with ACD15 and ACD21

**Figure 1:**
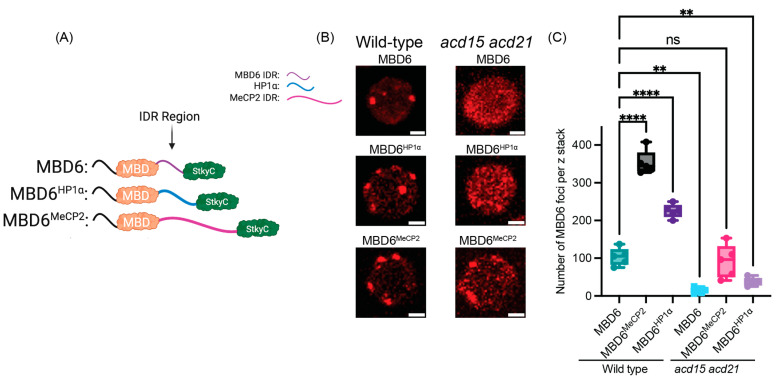
IDRs can enhance MBD6 nuclear foci. (**A**) Cartoon schematic of MBD6 IDR chimeric proteins, made using BioRender.com. (**B**) Representative root nuclei demonstrating localization patterns of MBD6 or MBD6-IDR fusion proteins in either wild-type or *acd15 acd21* mutant plants. Scale bar = 2 μM. (**C**) MBD6 nuclear foci counts across z-stacks of root tissue from multiple plant lines (N = 5 for each construct tested) in both wild-type and *acd15 acd21* mutant plants. Statistically compared using one-way ANOVA with multi-comparisons using Dunnett’s test. (*p* values: **** < 0.0001, ** < 0.01, ns >= 0.05).

MBD6 contains an IDR region between its MBD and StkyC domains, which is dispensable for function and protein accumulation in vivo [11]. To determine if IDRs that are known to induce strong multimerization could contribute to MBD6 protein accumulation, we replaced the natural IDR of MBD6 (amino acids 149–167) with the amino acid sequence of two human IDRs: the hinge domain of HP1α (amino acids 70–117) and the second IDR region of MeCP2 (amino acids 167–486) (Figure 1A) [25,26]. These IDRs vary in both the size and number of charged residues, but both IDRs induce strong multimerization in human and mouse cells, leading to the establishment of protein accumulation in chromatin (Figure S1A) [25,26,27,28]. To control the amount of protein produced and avoid overexpression artifacts, MBD6-IDR chimeric proteins were expressed using the endogenous MBD6 promoter and contained C-terminal RFP tags for live cell imaging. 

Wild-type MBD6 consistently forms nuclear foci in root cells, localizing to chromocenters, and this process depends on binding to methylated DNA through the MBD domain and on interaction with ACD15/ACD21 to accumulate MBD6 and its complex members [11,14]. Remarkably, we found that the replacement of the natural IDR of MBD6 with the IDRs of HP1α (MBD6^HP1α^) or MeCP2 (MBD6^MeCP2^) greatly increased MBD6 chromocenter localization as compared to wild-type MBD6, as quantified by the number of nuclear foci visualized in the root cells (Figure 1BC and Figure S1B). However, when the MBD6-IDR constructs were expressed in *acd15 acd21* mutant plants, the increase in chromocenter localization was greatly diminished, with MBD6^HP1α^ showing a complete lack of nuclear foci and MBD6^MeCP2^ showing a reduced number of nuclear foci (Figure 1B,C). The IDR of MeCP2 is over six times the size of the HP1α IDR, which is likely to provide more opportunities for multivalent interactions and the multimerization of complexes containing MBD6^MeCP2^ proteins, possibly explaining its ability to create more foci than MBD6^HP1α^. Notably, the number of MBD6^MeCP2^ foci formed in *acd15 acd21* mutant plants was roughly similar to the number of foci formed by wild-type MBD6, suggesting that an IDR can replace the role of ACD15 and ACD21 in MBD6 chromocenter accumulation (Figure 1C). 

Because the two tested IDRs were of human origin, we also sought to test whether an endogenous plant IDR, not related to the MBD5/6 complex, could also drive the accumulation of MBD6. Therefore, we tested whether the IDR from the Arabidopsis linker histone H1.1 could also induce the accumulation of MBD6 at chromocenters and substitute for the function of ACD15 and ACD21. The C-terminal IDR of H1.1 was recently shown to be important in mediating H1.1 accumulation and forming phase-separated condensates at chromocenters similar to MBD6 [29]. Like the human IDRs, we again swapped the natural IDR of MBD6 with the amino acid sequence of the C-terminal IDR of H1.1 (amino acid 130–274), which is three times the size of the HP1α IDR (Figure S1A). Similar to the human IDR fusion constructs, MBD6^H1.1^ hyper-localized to chromocenters in the wild-type cells, forming nuclear foci with almost no diffuse signal in the nucleoplasm (Figure S1B,C). In addition, like MBD6^MeCP2^, MBD6^H1.1^ retained nuclear foci formation in the *acd15 acd21* mutant background, demonstrating that the H1.1 IDR could also substitute for the role of ACD15 and ACD21 in driving the accumulation of MBD6 at the chromocenters (Figure S1C). To control for the possibility that the increase in foci could be caused by excess protein, we also measured the RFP signal of individual wild-type nuclei from plants expressing MBD6, MBD6^HP1α^, MBD6^MeCP2^, and MBD6^H1.1^. We found no significant differences between nuclear intensities, suggesting that the foci trends of MBD6-IDR constructs are not caused by differences in the amount of MBD6 in these nuclei (Figure S1D). 

**Figure 2:**
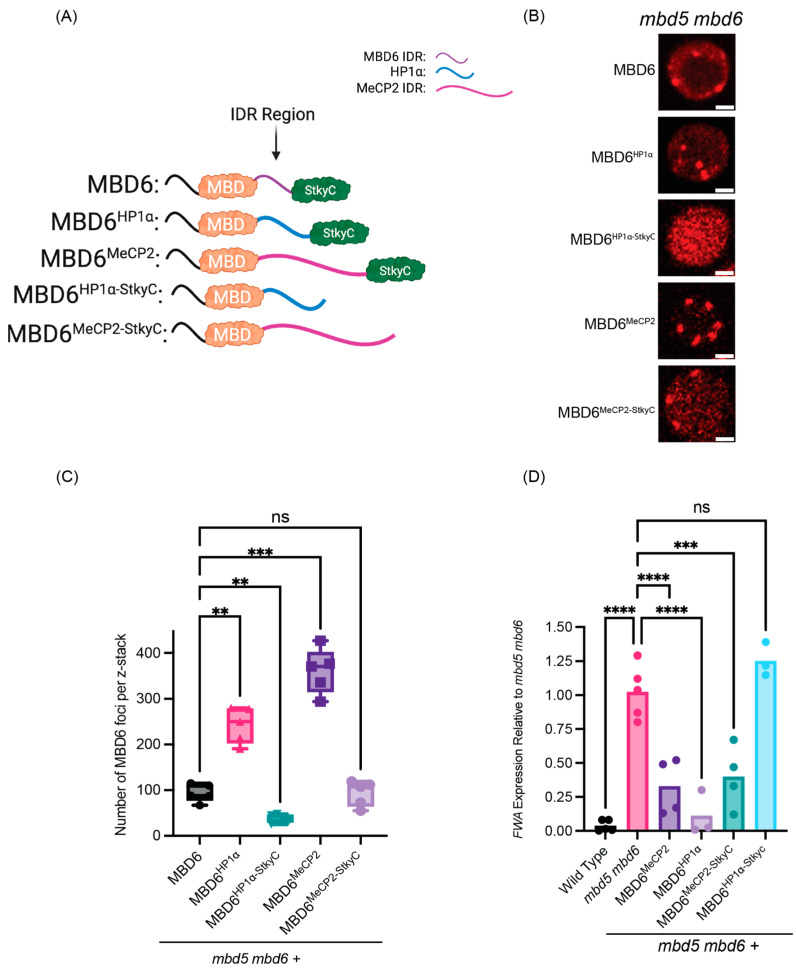
Synthetically accumulating MBD6 can mimic gene silencing. (**A**) Cartoon schematic of MBD6 IDR chimeric proteins, made using BioRender.com. (**B**) Representative image of root nuclei from *mbd5 mbd6* mutant plants expressing MBD6 or MBD6-IDR fusion proteins. Scale bar = 2 μM. (**C**) Foci counts across the z-stack of *mbd5 mbd6* mutant plants expressing either MBD6 or MBD6-IDR fusion proteins (N = 5 plants per construct). Statistically compared using one-way ANOVA with multiple comparisons using Dunnett’s comparison test. (*p*-value: *** < 0.001, ** < 0.01, ns >= 0.05). (**D**) *FWA* expression normalized to the *mbd5 mbd6* mutant background from RT-qPCR. Statistically compared using one-way ANOVA with multiple comparisons using Dunnett’s comparison test. (*p*-value: **** < 0.0001, *** < 0.001, ns >= 0.05).

To further confirm the combinatorial effect of IDR- and ACD-mediated accumulation on MBD6, we expressed HP1α and MeCP2 MBD6 chimeric proteins lacking the StkyC domain that is needed for the interaction of MBD6 with ACD15 (MBD6^HP1α-StkyC^ and MBD6^MeCP2-StkyC^) and compared these constructs with MBD6^HP1α^ and MBD6^MeCP2^ in the *mbd5 mbd6* mutant background (Figure 2A) [11]. MBD6^HP1α^ and MBD6^MeCP2^ again demonstrated a greatly increased number of nuclear foci as compared to wild-type MBD6. However, MBD6^HP1α-StkyC^ was unable to create nuclear foci, while the number of MBD6^MeCP2-StkyC^ foci significantly decreased as compared to MBD6^MeCP2^ (Figure 2B,C). These nuclear phenotypes were further characterized by plotting the nuclear distribution of the RFP signal for the MBD6-IDR fusion constructs across root nuclei, which again highlighted the dramatic difference between MBD6^HP1α^ and MBD6^HP1α-StkyC^, while the difference between MBD6^MeCP2^ and MBD6^MeCP2-StkyC^ was less pronounced (Figure S2A–D). We also verified the decrease in chromocenter localization by staining for DNA using 4′,6-diamidino-2-phenylindole (DAPI), which confirmed that MBD6^HP1α^ overlaps with chromocenters while MBD6^HP1α-StkyC^ does not (Figure S2E).

These results demonstrate that IDRs can synthetically induce MBD6 to hyperaccumulate at chromocenters, with the IDRs of MeCP2 and H1.1 substantially substituting for the roles of ACD15 and ACD21 as mediators of MBD6 accumulation. This suggests that the mechanisms by which α-crystalline domains and intrinsically disordered regions accumulate proteins share similarities, leading to parallel phenotypic outcomes.

### 2.2. IDR Accumulation of MBD6 Is Associated with Gene Silencing

We showed previously that MBD6 chromocenter accumulation correlates with gene silencing by the MBD5/6 complex, wherein the losses of ACD15 and ACD21 lead to an inability of the MBD5/6 complex to accumulate or silence genes and TEs [11]. We therefore hypothesized that MBD6-IDR chimeric proteins might replicate the gene-silencing function of MBD6 if MBD6-IDR proteins can sufficiently substitute for the role of ACD15 and ACD21 in accumulating MBD6 at methylated sites (Figure 1B,C and Figure 2B,C). *FWA* is a promoter-methylated and silenced target of the MBD5/6 complex, which becomes derepressed in *mbd5 mbd6* mutant plants, a phenotype that can be complemented upon the expression of wild-type MBD6-RFP [11,14]. We therefore utilized *FWA* expression to measure the ability of MBD6-IDR fusion proteins to complement the gene-silencing function of MBD6 in *mbd5 mbd6* mutant plants. 

Using reverse transcription with quantitative PCR (RT-qPCR) from the RNA of flower buds, we found that MBD6^HP1α^ and MBD6^MeCP2^ were able to significantly reduce the expression of *FWA* in *mbd5 mbd6* plants (Figure 2D). However, MBD6^HP1α-StkyC^, which was unable to form nuclear foci, led to no reduction in the *FWA* expression, showing that its silencing function was abolished (Figure 2B–D). On the other hand, MBD6^MeCP2-StkyC^, which retained the ability to form nuclear foci, was able to significantly reduce *FWA* expression (Figure 2B–D). These gene expression results correlate with foci formation data, suggesting that gene silencing by MBD6 is at least in part dictated by efficient accumulation at methylated sites regardless of whether that accumulation is induced by ACD15/ACD21 or by IDRs. 

## 3. Conclusions

**Figure 3:**
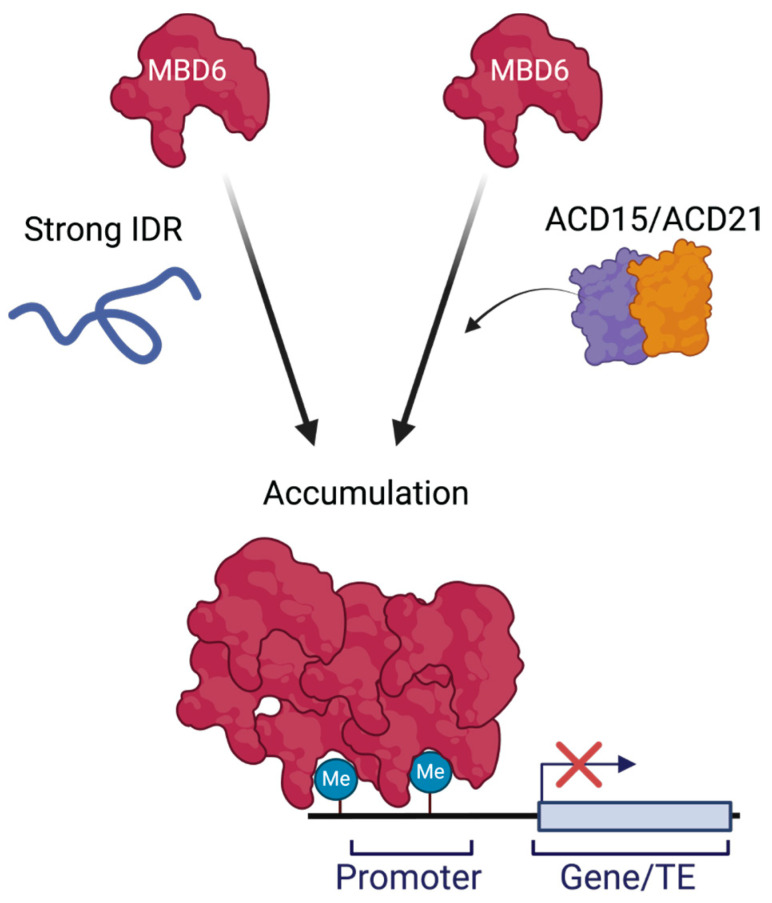
Model of MBD6 accumulation at methylated sites through endogenous and synthetic mechanisms (IDRs). The DNA methylation-binding protein, MBD6, can be synthetically hyper-accumulated using IDRs. However, ACD15 and ACD21 also control protein accumulation and can work together with these IDRs to maintain efficient MBD6 localization. Therefore, both mechanisms can functionally drive MBD6 accumulation. Made using BioRender.

Our results demonstrate that IDRs can synthetically drive the accumulation of MBD6 to chromocenters additively with ACD15 and ACD21, and that, surprisingly, they can overcome the need for ACD15 and ACD21 in some cases. The IDRs of HP1α, MeCP2, and H1.1 are all naturally required for those respective proteins to accumulate and form phase-separated compartments in cells [25,26,29]. When added to MBD6, these IDRs mimicked the function of ACD15 and ACD21, likely acting as sites for protein-protein interactions between MBD6 proteins. The accumulation of MBD6 at chromocenters thus can be determined by the ability of MBD6 to form supramolecular assemblies, whether driven by ACDs or by IDRs (Figure 3. These results also suggest that the phase separation models proposed for HP1α, MeCP2, and H1.1 are likely operative in these MBD6-IDR chimeric proteins. Still, it is remarkable that the IDRs from human HP1α and MeCP2, both related to heterochromatin formation and gene silencing in humans, function in the context of plant heterochromatin. This suggests that IDRs may retain protein accumulation functions regardless of the organism in which they are utilized. 

Our results also further highlight the importance of protein accumulation for gene silencing by the MBD5/6 complex. Interestingly, MeCP2 can maintain some level of gene-silencing function even without the StkyC domain. This suggests that protein accumulation of MBD6 alone can silence genes in Arabidopsis to a certain extent. However, because MBD6^MeCP2^ and MBD6^MeCP2-StkyC^ silence *FWA* equally but not fully, this suggests that IDRs are not able to fully recapitulate the gene-silencing roles of ACD15 and ACD21 in the MBD5/6 complex. We suggest the possibility that a specific amount or quality of the accumulation is needed for gene silencing to happen efficiently. The additive nature of MBD6 accumulation via both ACDs and IDRs also emphasizes that the IDR- and ACD-mediated protein accumulations are likely mechanistically related and can work simultaneously in the same complex. 

It is important to highlight the primary use of an ACD-mediated mechanism to accumulate the MBD5/6 complex. We hypothesize that plants may have needed an efficient, organized, and regulated mechanism for protein accumulation and thus coopted small heat-shock proteins for this purpose. These findings are consistent with many early studies showing that sHSPs from different organisms form dynamic oligomeric complexes [16,17,22] and with more recent studies showing that some sHSPs are linked to the formation and regulation of phase separation events created by proteins utilizing IDRs for accumulation [30,31,32]. Finally, our results further confirm and highlight that the multimerization and hyperaccumulation of MBD6 at DNA-methylated sites in heterochromatin are critical aspects of its gene-silencing function.

## 4. Materials and Methods

### 4.1. Plant Materials and Growth Conditions

All plants used in this study were in the Columbia-0 ecotype (Col-0). The plants were grown on soil in a greenhouse under long-day conditions (16h light/8h dark). The plants grown for microscopy were plated on 1/2xMS plates in growth rooms at room temperature (~25 °C), with 16h of light and 8h of dark. 

The following mutant lines were previously described: *mbd5 mbd6* T-DNA double mutant composed of mbd5 T-DNA line SAILseq_750_A09.1 and mbd6 T-DNA line SALK_043927. The *acd15 acd21* mutant plant line was created using CRISPR-Cas9 editing as described previously [11].

### 4.2. Generation of Transgenic Lines

Transgenic plants expressing fluorescently tagged proteins were created using the pGWB553 destination vector obtained from Addgene. Specifically, the MBD6 promoter and coding sequences were PCR-amplified from genomic DNA and cloned into pENTR/D-TOPO vectors. These coding sequences were then inserted into the final destination vector (pGWB553) using the Gateway LR Clonase II Enzyme mix (Catalog number: 11791020, ThermoFisher, Los Angeles, CA, USA). These destination vectors were electroporated into AGL0 agrobacterium and transformed into Col0 (wild-type), *mbd5 mbd6* (SALK_043927), and *acd15 acd21* plants. The positive selection of transgenic plants was done on ½ MS agar plates with hygromycin B after 5 days in the dark at 4 °C, 8 h in the light at room temperature, and another 5 days in the dark at room temperature. All protein expression constructs in this paper are RFP tagged. 

### 4.3. Confocal Microscopy

All confocal microscopy experiments were performed using the Zeiss, LSM 980 confocal microscope, Jena, Germany. Unless otherwise stated, all experiments were performed using a 40× magnification water objective lens. 

Live plant samples were prepared for microscopy as follows:

Two-week-old seedlings were grown on ½ MS plates at room temperature, ~25 °C, and transferred onto 1mm-thick glass slides (Fisherscientific, Los Angeles, CA, USA, Cat No. 12-550-08) containing deionized water (room temperature). The seedlings were oriented such that the roots were on the middle of the slide while leaves extended from the top of the slides. On top of the plant were placed #1.5 coverslips (Fisherscientific, Cat No. 12-544-EP).

### 4.4. Quantification of Foci Counts and Nuclear RFP Signal Distributions

All foci counts and nuclear distribution plots were quantified using ImageJ, image analysis software (https://imagej.net/ (accessed on 22 August 2024)).

The nuclear intensities were measured using Zeiss, Zen Blue software (version 3.10). The nuclei were circled using the same diameter across the nuclei (8 μM), and the intensities were measured using the “measure” program of the Zeiss software (https://www.zeiss.com/microscopy/en/products/software/zeiss-zen.html (accessed on 22 August 2024)). These intensities were then plotted and compared using GraphPad Prism software (https://www.graphpad.com/ (accessed on 22 August 2024)).

A plot profile macro was used to obtain measurements of the RFP intensity of MBD6-IDR constructs across the nuclei for Supplemental Figure S2. The RFP intensity of each MBD6-IDR construct was measured across the red line shown in the nuclear images at the widest diameter of each nucleus.

Foci counts were obtained using the 3D object counter app in ImageJ software. Foci counts were obtained from 50-slice z-stacks of the root meristems for each plant imaged (N = 5 for each specific construct). The 3D objects counter app in ImageJ uses an arbitrary numbering system for each image to establish a fluorescence signal threshold to allow the software to recognize 3D objects. To maintain consistent thresholding, the same signal threshold was used across all images.

All z-stack images of roots used in this study were the same depth through the root across all replicates and used the same imaging settings (i.e., magnification and laser intensity). Foci counts and nuclear distribution intensity values were all plotted using GraphPad Prism software, and statistical analysis was performed using GraphPad Prism software as mentioned in figure legends.

### 4.5. RT-qPCR

All RT-qPCR experiments were performed on cDNA created from the RNA of unopened floral bud tissue. The RNA was extracted using the Zymo Direct-zol RNA MiniPrep Kit (Zymo Research, R2052, Los Angeles, CA, USA). Approximately 400 ng of the total RNA was reverse-transcribed into cDNA with Superscript III First Strand Synthesis Supermix (Invitrogen, Los Angeles, CA, USA, 18080400), using random hexamers. The qPCR was performed with iQ SYBR Green Supermix (Bio-Rad, Los Angeles, CA, USA, 1708882) with the Agilent Technologies Mx3005p qPCR System (Stratagene, Los Angeles, CA, USA). A total of 0.5 µL of cDNA was used for each 20 µL reaction, with technical triplicates for each primer pair. The housekeeping gene *ISOPENTENYL PYROPHOSPHATE DIMETHYLALLYL PYROPHOSPHATE ISOMERASE 2* (*IPP2*) was used as a control. The analysis of the qPCR curves was performed using BioRad qPCR software (https://www.bio-rad.com/zh-cn/category/image-lab-software-resources?ID=PJWA0VTU86LJ (accessed on 22 August 2024)) with the expression level of *FWA,* calculated as ΔΔC_t_ as compared to the expression of the *IPP2* control gene. A statistical analysis of the *FWA* expression of *mbd5 mbd6* plants expressing the MBD6-IDR constructs was performed using GraphPad Prism, and specific statical analyses are indicated in the figure legends.

List of primers used for RT-qPCR:

*FWA* RT-qPCR Forward: TTAGATCCAAAGGAGTATCAAAG
*FWA* RT-qPCR Reverse: CTTTGGTACCAGCGGAGA
*IPP2* RT-qPCR Forward: GTATGAGTTGCTTCTCCAGCAAAG
*IPP2* RT-qPCR Reverse: GAGGATGGCTGCAACAAGTGT

## Data Availability

All data are available within this manuscript.

## Supplementary Materials

The following supporting information can be downloaded at https://www.mdpi.com/article/10.3390/epigenomes8030033/s1: Figure S1: IDRs can hyperaccumulate MBD6 in root cells; Figure S2: MBD6 = IDR fusion proteins require StkyC domain for hyperaccumulation.
