# Supplementary material for: α-Crystalline Domains and Intrinsically Disordered Regions Can Work in Parallel to Induce Accumulation of MBD6 at Chromocenters in Arabidopsis thaliana
Source: Epigenomes. 2024 Aug 28;8(3):33. doi: 10.3390/epigenomes8030033 (PMC11417779; doi:10.3390/epigenomes8030033)
Supplement: Supplementary file 1 [file epigenomes-08-00033-s001.zip › epigenomes-3100457-supplementary.pdf]

Supplementary Figures

A)

IDR	Size	Positive Charge Residues	Negative Charge Residues
H1.1	145 AA	48	2
MeCP2	321 AA	68	35
HP1α	48 AA	15	8

C)

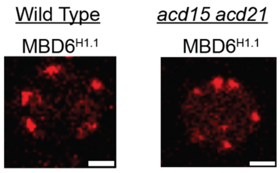

B)

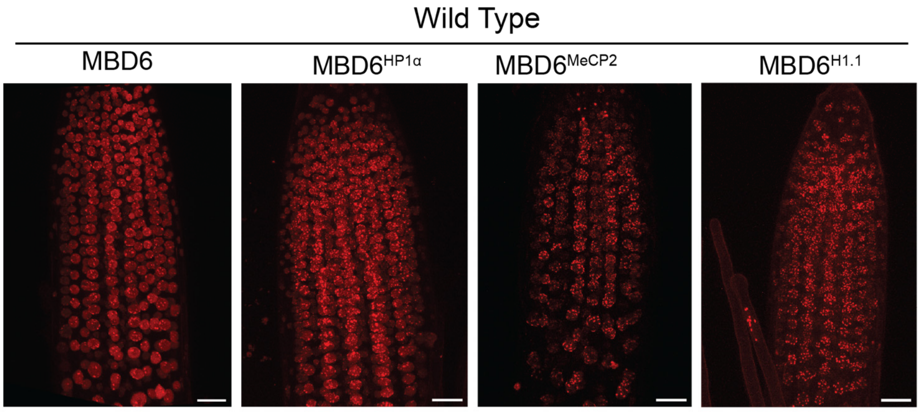

Figure S1. IDRs can hyperaccumulate MBD6 in root cells.
(**A)** Table comparing the size and charge composition of the amino acid sequences of three IDRs used in this study. **(B)** 3D reconstruction of root tips from z-stacks of wild-type plants expressing either MBD6 or MBD-IDR fusion proteins. The scale bar is 20μM. **(C)** Representative root nuclei comparing MBD6$^{H1.1}$ in wild type or acd15 acd21 mutant plants.

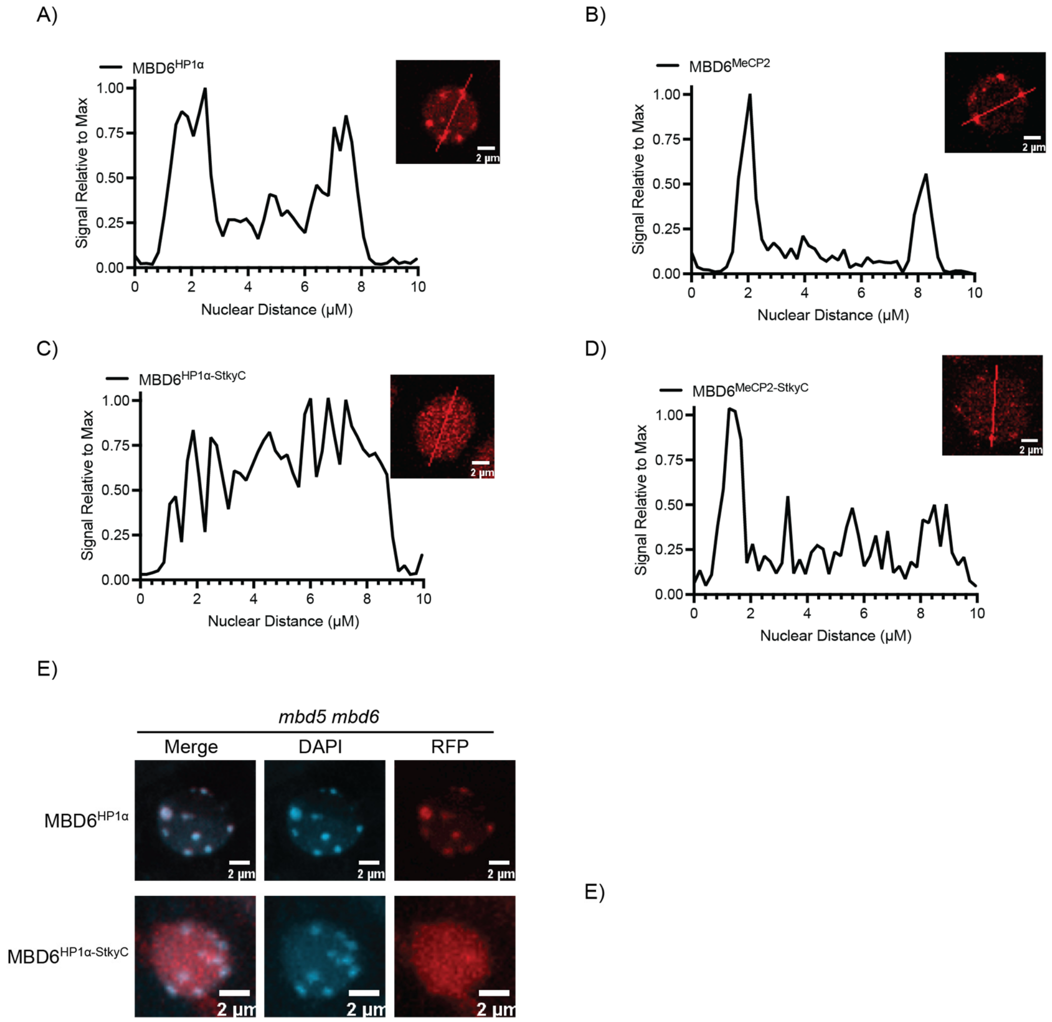

Figure S2. MBD6-IDR fusion proteins require StkyC domain for hyperaccumulation.
(A-D) RFP signal of MBD6-IDR fusion proteins plotted along red line imposed across the images of root cell nuclei. The RFP signal plotted in each graph is normalized to the maximum intensity detected for the specific MBD6-IDR fusion protein tested in each plot.
(E) Representative root cell nuclei of MBD6$^{HP1\alpha}$ with and without the StkyC domain stained with DAPI to reveal the chromocenters.
